# Information system support for case-based knowledge formation in social welfare: a cross-sectional study

**DOI:** 10.1177/18333583251343681

**Published:** 2025-06-17

**Authors:** Elina Tynkkynen, Samuel Salovaara, Johanna Viitanen, Tinja Lääveri

**Affiliations:** 1 University of Jyväskylä, Finland; 2 University of Lapland, Finland; 3 Aalto University, Finland; 4 University of Helsinki and Helsinki University Hospital, Finland

**Keywords:** technology, informatics, evaluation, health information management, usability, user experience, social work, client information system, national survey

## Abstract

**Background:** Although client information systems (CIS) should provide social welfare professionals (SWPs) with a comprehensive overview of a client’s situation for case-based knowledge formation (CBKF), research into SWPs’ user experiences is scarce. **Objective**: The aim of this study was to examine SWPs’ experiences of CISs’ support for CBKF. **Methods:** In 2020, a nationwide cross-sectional CIS usability survey was conducted with 980 respondents in Finland. The 16 questionnaire statements pertained to technical functionality, usability, client information quality and access to case-based information in CISs. The factors contributing to CISs’ support for CBKF were analysed using univariable and multiple classification analyses. **Results:** The strongest predictors of CIS support for CBKF were usability and quality of information. Moreover, SWPs working in institutional care were more satisfied than their colleagues in other working environments. **Conclusion**: SWPs perceived the CISs’ information quality to be good, but there was a need for improvement in usability, for instance, via comprehensive summary views and dashboards of essential information. **Implications for health information management practice**: The findings highlight that while the information quality of CISs is generally perceived as good, improving usability is crucial for enhancing support for CBKF among SWPs.

## Introduction

Client information systems (CIS) have become essential tools for social welfare professionals (SWPs), enabling documentation, client information management and decision-making (Barrera-Algarín et al., 2021; Fitch, 2019; Huuskonen and Vakkari, 2015; Salovaara and Ylönen, 2022; Zhu and Andersen, 2021). Further, CISs support SWPs in forming a comprehensive understanding of a client’s situation, managing case information and delivering personalised services (Huuskonen and Vakkari, 2013). Case-based knowledge formation (CBKF) refers to the process of understanding a client’s situation within their social context, essential for effective practice (Salovaara and Ylönen, 2022). Rooted in foundational theories such as Mary Richmond’s early work on the relationship between individuals and their environments (e.g. Richmond, 1922), CBKF emphasises assessing a client’s circumstances, including case history, social networks and services (Hall et al., 2010; Huuskonen and Vakkari, 2015; Pithouse et al., 2012; Räsänen, 2012; Wastell and White, 2014). It integrates theoretical, factual and practical knowledge into a structured understanding that guides interventions and decisions (Trevithick, 2008). This dynamic, collaborative process requires professionals to continuously gather, analyse and synthesise information with clients, kinship networks and other professionals (Bronstein, 2003; Pohjola and Korhonen, 2014; Yliruka et al., 2018). The evolving understanding ensures timely, relevant decisions as situations change. Collected information supports decision-making and documentation, including the client plan (Fitch, 2019; Salovaara et al., 2022b, 2022c), which compiles the client’s history, social network, challenges, strengths, support needs, goals and strategies to achieve those goals (Finlex, 2014).

The development of CISs has mostly been driven by administrative needs and organisations’ demands to enhance work and services with the help of analysable data (De Corte et al., 2019; Devlieghere and Roose, 2019; Seniutis et al., 2021). Finland implemented the first nationally uniform structured classification of social work in 2000 with the aim to support documentation in a structured and standardised format in a CIS, improve service quality and increase the utilisation and sharing of client information through the systems (Kallinen-Kräkin, 2001). However, as documentation of social work has traditionally relied on free-text narratives (Devlieghere and Roose, 2018; De Witte et al., 2016), SWPs may feel that information systems hinder their familiar habit of documentation (Parton, 2009; Pithouse et al., 2012; Räsänen, 2012; De Witte et al., 2016), for example, by requiring the use of predefined headings, checkboxes, or dropdown menus (Gillingham, 2014b). As a result, SWPs have reported decreased information quality and increased difficulty in forming and understanding cases (Pithouse et al., 2012; Salovaara and Ylönen, 2022; Seniutis et al., 2021).

When assessing the usefulness and success of information systems from the end users’ viewpoint, utility, usability and user experience are key concepts. According to Nielsen (2012) utility refers to whether the necessary features exist in the system, whereas usability relates to the interaction between the user and the interactive system and can be examined through various attributes such as efficiency, learnability, error rate and user satisfaction. User experience emphasises the user’s holistic experience of the interactive system, including the user’s expectations, perceptions and experiences before, during and after use (Law et al., 2009). Further, the Delone and McLean IS success model examines how system quality, information quality and service quality influence user satisfaction and the use of ISs (DeLone and McLean, 1992, 2003).

The interest in usability and the user experience of CISs in social welfare has grown in recent years, but research is still scarce (Devlieghere and Roose, 2019; Gillingham, 2014a; Gillingham and Graham, 2016). Previous international studies on CISs have mostly applied qualitative research approaches and focused on a particular service line or included a small research sample (Ylönen, 2022). The few Finnish studies focusing on the usability of CISs have found that CISs do not provide support for SWPs’ key tasks (Salovaara and Ylönen, 2022; Salovaara et al., 2022c; Ylönen et al., 2020) and, internationally, needs of frontline professionals and management regarding CISs are sometimes in conflict with each other (De Corte et al., 2019; Devlieghere and Roose, 2019; Seniutis et al., 2021). Studies on the user experience of CISs have identified inadequacies in usability and systems’ support for providing an overview of the client’s situation (Salovaara et al., 2022c; Ylönen et al., 2020). However, little attention has been given to CISs’ support for CBKF in social welfare (Salovaara and Ylönen, 2022). Currently, CISs are used more like typewriters, and SWPs accustomed to handling narrative text use various – seemingly illogical – classifications for the input of data that end users often find irrelevant (Lagsten and Andersson, 2018). It is not enough that the documented information is available; it should also be easily accessible and useful for SWPs in their daily work with clients. Well-functioning CISs can also support the general well-being of health and social care professionals (Alobayli et al., 2023; Heponiemi et al., 2019; Nadav et al., 2024; Nguyen et al., 2021).

### Context of the study

In Finland, CISs have been used in social welfare since the 1970s (Kuusisto-Niemi, 1999). By 2014, nearly all public sector organisations were using CISs (Kärki and Ryhänen, 2015); however, as of 2020, approximately a quarter of private sector and non-governmental organisations (NGOs) still lacked information systems (Salovaara et al., 2022b). The public social welfare sector focuses on preventing social problems, maintaining social security and supporting independent living by providing various social services from child welfare to elderly care, assistance, loans, guidance and counselling (Finlex, 2014). Public service organisers also purchase social services from private providers and NGOs (Vehko, 2022). Most social workers (95%) and social counsellors (56%) are employed by public sector municipalities (Vuorijärvi et al., 2019); in the private sector and NGOs, most employees are practical nurses (Tilastoraportti, 2023). In the public sector, professionals manage client situations end-to-end and provide various services, requiring comprehensive functionalities from information systems. Private sector and NGOs typically are service providers, often offering only one service or specialising in a specific client group, which is why they need more focused CISs. Large systems are often more complex, while focused systems tend to be simpler (Gillingham, 2019). Some social services, such as home care, are based on health issues, so SWPs may use electronic health records in their work.

Since the 2010s, information management development has expanded to cover all social services and unified structures have been developed to harmonise data management across different specialties or service lines and to support the interoperability of CISs (Ailio and Kärki, 2013). National-level efforts in social welfare services have focused on the development of a national client data repository, which aims to provide easy and secure access to social welfare client data for professionals (Kuusisto-Niemi et al., 2018; Vehko, 2022). Finnish national legislation strictly regulates information sharing; clients’ specific consent is usually needed to share data, even between professionals working within the same social welfare organisation (Vehko, 2022). National architecture also defines which client data the professionals can access. In addition, social welfare and healthcare data are stored in separate registries and specific consent is required to share information between SWPs and healthcare professionals, for example, in elderly or substance abuse services (Vehko, 2022).

### Aim of the study

The aim of this study was to explore SWPs’ experiences of CISs’ support for CBKF, which is crucial from the perspective of professionals’ routine work and has not been widely studied. The research questions were:

• What kind of experiences do SWPs have regarding CISs’ usability, information quality and technical quality?
• How do CISs support SWPs in CBKF?
• What factors contribute to CISs’ support for CBKF?

## Methods

### Questionnaire and data collection

The large national survey data for this study were gathered in Finland during October and November 2020 (Salovaara et al., 2022a). The survey was based on a 2019 pilot study (Ylönen et al., 2020) that utilised validated usability variables (Hyppönen et al., 2019) originally designed for physicians (Vänskä et al., 2010). The statements for this study were updated based on insights from the pilot study, and the survey was tested and finalised with input from social welfare experts (Salovaara et al., 2022a). The questionnaire is available online in Finnish (Sote-digitalisaation seurantatutkimuksen toteuttaminen, 2024).

The data were collected by the University of Lapland as part of a national research project coordinated by the Finnish Institute for Health and Welfare. Data collection was conducted using an online tool distributed via email links in collaboration with the Talentia Union of Professional Social Workers, the Trade Union for the Public and Welfare Sectors, and the Social Work Research Society. When it became apparent that the survey did not reach enough intended respondents (due to, for example, incomplete contact information), the response period was extended and the invitation was disseminated more broadly through social welfare networks, including Centres of Expertise on Social Welfare (Salovaara et al., 2022a). The precise response rate could not be calculated because information on the exact number of recipients to whom the survey was successfully delivered was not available, but there were, in total, over 12,000 members in those unions at that time.

### Statistical analysis

From the questionnaire, we selected 16 statements pertaining to technical functionality, usability, client information quality, and access to case-based information in CISs for this study (Box 1). Statistical analyses were conducted using IBM SPSS Statistics version 28 (Armonk, New York, United States). Four summary variables were created from individual variables based on the dimensions of usability (Nielsen, 2012), the IS success model (DeLone and McLean, 1992, 2003), previous NuHISS research (Hyppönen et al., 2019), and CISs’ support for CBKF (Salovaara and Ylönen, 2022). All summary variables were tested with Cronbach’s alphas (0.764, 0.811, 0.822 and 0.722, respectively) (Taber, 2018). For cross-tabulation and multiple classification analysis, the response scale of individual statements of usability, information quality, technical quality and CBKF was reclassified on a scale from 1 to 5, with the weakest response assigned a value of 1 and the strongest response assigned a value of 5. After that, summary variables for usability, information quality and technical quality were combined into a dichotomous scale as good (value of 3.01 or more) or poor (value of 3.00 or less). The statement for CIS support for forming CBK was reclassified, and because we did not want to classify that as dichotomic, we transformed it into a three-level scale: good, (3.31 or higher), neither good nor poor (2.70–3.30) and poor (2.69 or lower).

**Box 1.** Questions and statements selected for analysis.

Question	Statement
Assess the technical functionality of the (your primarily used^a^) CIS in (your working environment^a^) from the perspective of an SWP, using the following statements.^b^	CIS is technically stable (no crashes, no use breaks).
	CIS responds quickly to commands.
Assess the usability of the (your primarily used^a^) CIS in (your working environment^a^) from the perspective of an SWP, using the following statements.^b^	Fields and functions in the views (windows) are logically arranged.
	Terminology (e.g. names of functions and headings) is clear and understandable.
	Performing routine tasks is straightforward and can be done without unnecessary choices.
	It is easy to correct made errors (such as error documenting or changing selections).
	CIS guides users to learn how to use the system.
Assess the quality of client information available from the (your primarily used^a^) CIS.^b^	Information is up-to-date.
	Information is reliable.
	Information is relevant.
	Information is covered and comprehensive.
Assess how easy or difficult it is with the support of the (your primarily used^a^) CIS to perceive following client-related information?^c^	Client’s multiprofessional network.
	Client’s social network.
	Client’s history.
	Client’s services (previous, current and future services).
	Client’s plan.

CIS: client information systems; SWP: social welfare professional.

a Pre-filled based on the respondent’s previous survey response.

b Answer options: *1 = Totally agree, 2 = Somewhat agree, 3 = Neither agree or disagree, 4 = Somewhat disagree, 5 = Totally disagree.*

c Answer options: *1 = Very easy, 2 = Quite easy, 3 = Neither easy or difficult, 4 = Quite difficult, 5 = Very difficult.*

Multiple classification analysis was used to determine how much an individual independent variable predicted CISs’ support for CBKF in a design where other independent variables were standardised. Independent variables were selected based on previous research and *p*-values (<0.001) from a chi-squared test.

### Ethic approval

The ethical approval was provided by the Finnish Institute for Health and Welfare institutional review board (THL482/6.02.01/2020).

## Results

### Respondent demographics

Of the 990 SWPs who responded to the survey, 980 were selected for this study because they were daily CIS users. Most respondents (86%) worked in the public sector and had over 1 year of experience using current CISs (84%) (Table 1). The reclassified demographic data for the multiple classification analysis are presented in the Supplemental Materials.

**Table 1.** Respondents’ characteristics.

Respondent characteristics		%	*n*
Age		100	979
	under 35	18.6	182
	35–44	35.0	343
	45–54	26.4	259
	over 54	20.0	195
Education		100	978
	Master’s degree or higher	51.9	508
	Bachelor’s degree or equivalent	48.1	470
Primary employment sector		100	980
	Public	85.6	839
	Private, association, other	14.4	141
Service line		100	980
	Child welfare	22.0	216
	Services for working-age people	16.7	164
	Services for disabled people	13.5	132
	Service for families with children	12.9	126
	Multiple service lines	12.0	118
	Other (i.e. in health care)	8.1	79
	Services for elderly people	5.7	56
	School	4.8	47
	Substance abuse services	2.2	22
	Family law services	2.0	20
Title		100	980
	Social worker	34.0	333
	Other	33.5	328
	Social counsellor	20.2	198
	Supervisor	12.3	121
Experience of use		100	970
	Under 1 year	16.2	157
	1 year or more	83.8	813

### Results of univariable analyses

**Figure 1.**
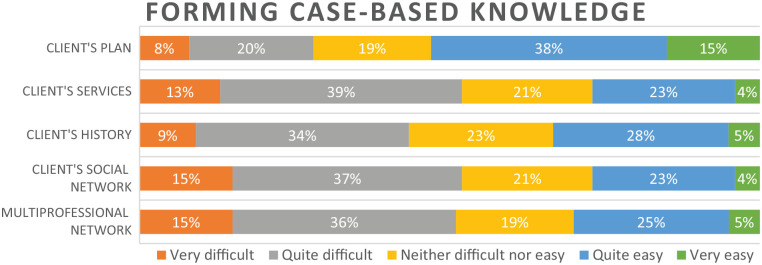
Responses to five statements concerning CISs’ support for CBKF. CIS: client information systems; CBKF: case-based knowledge formation.

**Figure 2.**
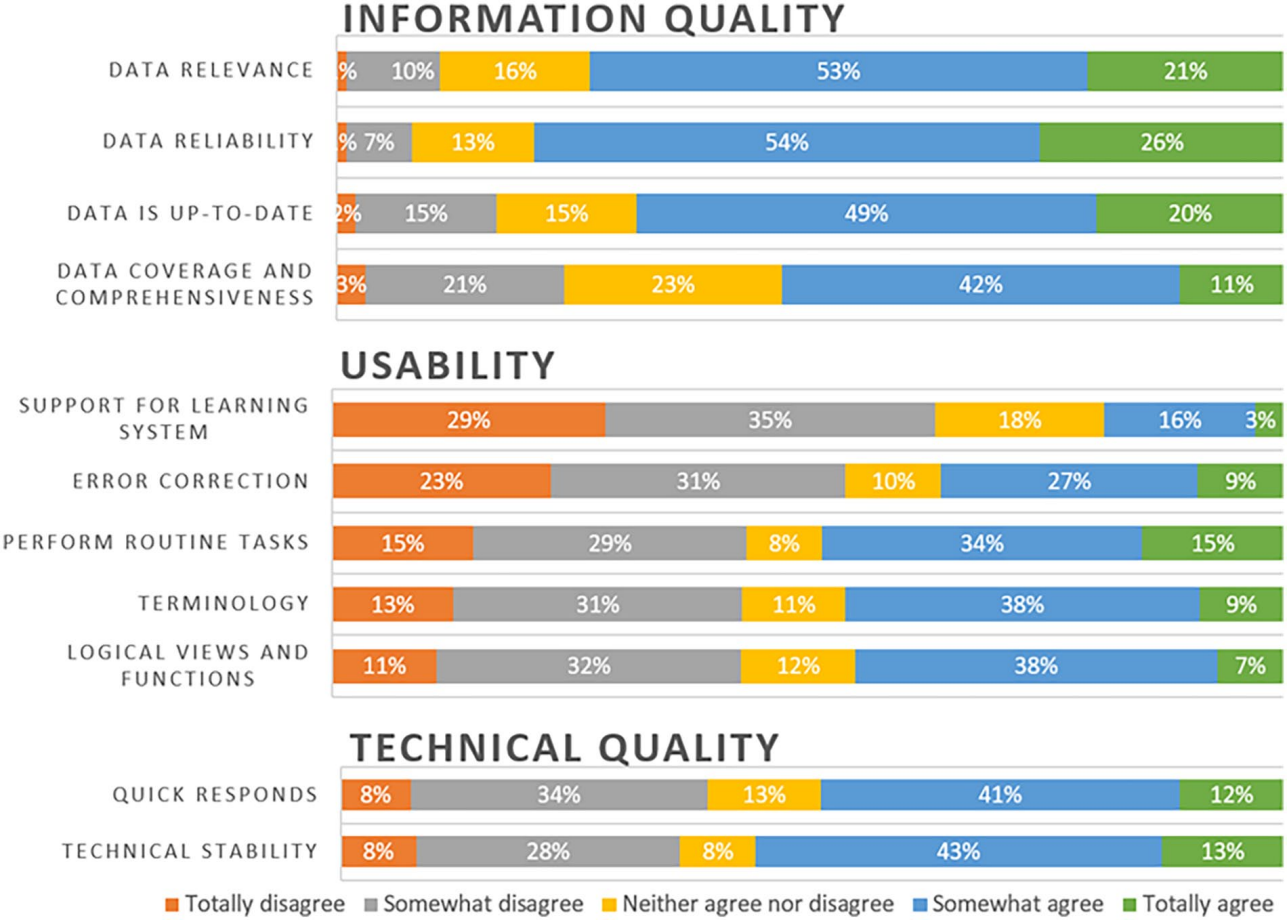
Responses to statements about usability, information quality and technical quality.

Half (53%) of the respondents assessed that it was easy (very or quite easy) to understand the client plan with the support of CISs (Figure 1). By contrast, half (43%) perceived forming an understanding of a client’s history as difficult. Experiences of CIS support for forming an understanding of a client’s services, social network and multiprofessional network were similar: 51–52% gave negative responses. Of usability statements, almost half of the respondents (45–49%) agreed that CISs support performing routine tasks, and that terminology, views and functions are logical (Figure 2). Most respondents (53–80%) agreed with the statements on information quality. Half of the respondents perceived CISs as technically stable (56% agreed) and responding quickly to commands (53% agreed).

The respondents’ primary employment sector and working environment, the type of CIS, usability, information quality and technical quality were associated with the ease of CBKF (Table 2). No associations were found for experience of CIS use, educational level, possible managerial position or service line. The reclassified working environment and type of CIS data for the multiple classification analysis are presented in the Supplemental Materials.

**Table 2.** Cross-tabulation, *p*-values and multiple classification analysis.

				CISs support for case-based knowledge *n*(%)				Multiple Classification Analysis					
			*N*	Poor	Neither poor nor good	Good	*p* value	Predicted Mean Unadjusted	Deviation unadjusted	Eta	Predicted Mean Adjusted for Factors	Deviation adjusted for Factors	Beta
Primary employment sector			961	416 (43.3)	271 (28.2)	274 (28.5)	<0.001***						
Public sector			828	378 (45.7)	231 (27.9)	219 (26.4)		2.80	−0.05		2.84	0.00	
Private, association, other			133	38 (28.6)	40 (30.1)	55 (41.4)		3.09	0.26		2.85	0.01	
										0.128			0.007
Professional’s working environment			961	416 (43.3)	271 (28.2)	274 (28.5)	<0.001***						
Open care services			685	307 (44.8)	187 (27.3)	191 (27.9)		2.83	−0.01		2.85	0.13	
Institutional services			147	40 (27.2)	50 (34.0)	57 (38.8)		3.08	0.24		2.98	0.14	
Health care			76	48 (63.2)	14 (18.4)	14 (18.4)		2.48	−0.36		2.53	−0.30	
Other			53	21 (39.6)	20 (37.7)	12 (22.6)		2.80	−0.04		2.72	−0.12	
										0.175			0.133***
CISs scope of use			959	415 (43.3)	271 (28.3)	273 (28.5)	<0.001***						
Large CIS			605	270 (44.6)	172 (28.4)	163 (26.9)		2.82	−0.02		2.87	0.04	
Focused CIS			129	32 (24.8)	41 (31.8)	56 (43.4)		3.17	0.34		2.87	0.03	
Electronic health record			165	90 (54.5)	43 (26.1)	32 (19.4)		2.59	−0.25		2.65	−0.19	
Other information system			60	23 (38.3)	15 (25.0)	22 (36.7)		2.99	0.16		2.93	0.10	
										0.207			0.109*
Technical quality			960	416 (43.3)	271 (28.2)	273 (28.4)	<0.001***						
Poor			468	239 (51.1)	133 (28.4)	96 (20.5)		2.66	−0.18		2.77	−0.07	
Good			492	177 (36.0)	138 (28)	177 (36.0)		3.01	0.17		2.90	0.06	
										0.221			0.082**
Usability			961	416 (43.3)	271 (28.2)	274 (28.5)	<0.001***						
Poor			597	324 (54.3)	176 (29.5)	97 (16.2)		2.60	0.24		2.66	−0.18	
Good			364	92 (25.3)	95 (26.1)	177 (48.6)		3.23	0.39		3.13	0.30	
										0.380			0.289***
Information quality			956	413 (43.2)	271 (28.3)	272 (28.5)	<0.001***						
Poor			214	150 (70.1)	43 (20.1)	21 (9.8)		2.34	−0.50		2.47	−0.37	
Good			742	263 (35.4)	228 (30.7)	251 (33.8)		2.98	0.14		2.94	0.11	
										0.335			0.248***
Working as supervisor			961	416 (43.3)	271 (28.2)	274 (28.5)	0.074	*R*2					0.249
Yes			164	58 (35.4)	51 (31.1)	55 (33.5)		*R*					0.499
No			797	358 44.9)	220 (27.6)	219 (27.5)		Grand mean					2.84
Experience of use			960	416 (43.3)	271 (28.2)	273 (28.4)	0.048						
Under 1 year			154	78 (50.6)	44 (28.6)	32 (20.8)							
1 year or more			806	338 (41.9)	227 (28.2)	241 (29.9)							
Education			959	414 (43.2)	271 (28.3)	274 (28.6)	0.167						
Master’s degree or higher			501	223 (44.5)	148 (29.5)	130 (25.9)							
Bachelor’s degree or equivalent			458	191 (41.7)	123 (26.3)	144 (31.4)							
Service line			961	416 (43.3)	271 (28.2)	274 (28.5)	0.038						
One service line			724	310 (42.8)	210 (29.0)	204 (28.2)							
Multiple service lines			115	40 (34.8)	34 (29.6)	41 (35.7)							
Other work in social welfare			122	66 (54.1)	27 (22.1)	29 (23.8)							

CIS: client information systems.

* *p* ⩽ 0.05. **p⩽ 0.01. ***p ⩽ 0.001.

### Multiple classification analysis for ease of CBKF

When comparing multiple independent variables, usability was the strongest predictive factor of CISs’ support for CBKF (beta coefficient = 0.289). For respondents who perceived usability as good, the predicted adjusted mean was 3.1, whereas for those who perceived it as poor, the mean was 2.7. The second strongest predictive factor for CBKF was information quality. In addition, the professionals’ working environment had an impact on CIS support for CBKF. Respondents in institutional care were most satisfied, whereas professionals working in health care or other environments gave the poorest assessments of how CIS supported them in CBKF. In the multiple classification analysis, the CIS type, technical quality or the primary employment sector did not predict SWPs’ experience of CIS support for CBKF.

## Discussion

CISs should help professionals by providing an overview and understanding of the client’s situation. This requires the availability of client information for information processing and management – such as supporting CBKF. Our study examined the support provided by information systems, focusing particularly on CIS usability and support for CBKF. User experiences were not affected by the length of system use. However, recently, before the time of the survey, a large-scale CIS was deployed in a municipality of 200,000 inhabitants.

### Usability and information quality associated with CIS support for CBKF

Our main finding was that usability was the strongest predictor for CISs’ support for CBKF. This concurs with previous studies that have found that usability improves knowledge-based management in social welfare (Salovaara et al., 2022c; Ylönen et al., 2020). In our study, SWPs did not experience CISs supporting routine tasks, and the system views were not considered logical. The CISs did not support easy correction of user errors, nor did they help the user learn how to use the systems. In healthcare, usability problems have been associated with work-related stress and frustration (Alobayli et al., 2023; Heponiemi et al., 2019; Nguyen et al., 2021). If client information is fragmented within the CIS, it is difficult to understand the client’s overall situation (Lagsten and Andersson, 2018; Salovaara and Ylönen, 2022). SWPs use workarounds and shadow documentation systems such as notes on spreadsheets to overcome problems with CISs, highlighting the need for practical solutions not available within the CISs (De Witte et al., 2016; Huuskonen and Vakkari, 2013; Wastell and White, 2014). Usability could be improved, for instance, with comprehensive summary views and dashboards of essential information. This further underlines the need for CIS usability improvements and human-centred development (International Organization for Standardization (ISO), 2019).

In the multiple classification analysis, professionals who perceived that the information offered by the CISs was up-to-date, reliable, relevant and comprehensive had a better experience with the systems’ support for CBKF compared to those who perceived the information quality as poor. This result is very understandable considering that the information available in CISs is relied upon to make important decisions in social welfare (Huuskonen and Vakkari, 2015; Räsänen, 2012). Timely and reliable information provides a more sustainable basis for decision-making. In general, the comprehensiveness and versatility of the information support SWPs’ understanding of the client’s overall situation (Gillingham, 2011; Hall et al., 2010; Salovaara and Ylönen, 2022).

Traditionally, SWPs have preferred to document client cases in a narrative format (Parton, 2008). However, it can be difficult to detect the core of the case among long narratives (Huuskonen and Vakkari, 2015). Discrete documentation can help SWPs and other professionals working with the client to better identify and understand relevant information. Respondents felt that the client plan enhanced the overview of the client’s situation. SWPs independently record client plans as standalone documents, typically entered into information systems in a free-text format, so this may have impacted professionals’ experiences.

In many countries where CISs have been widely used in social welfare, documentation is guided toward a more structured approach to data processing (Pithouse et al., 2012; Wastell and White, 2014; Zhu and Andersen, 2021). CISs have been used in Finnish social welfare since the 1970s (Kuusisto-Niemi, 1999), and they are strongly defined by mandatory national requirements and definitions (Jormanainen et al., 2022). Furthermore, a systematic and consistent data structure enables the further development of technological innovations (Jormanainen et al., 2022). The development of CISs in accordance with national-level requirements in recent years requires a great deal of resources from IS vendors (Jormanainen, 2018), which may, in part, have slowed down the development of system usability. Our results confirm that research on the usability of CISs remains important in order to support CIS development and improve the professionals’ experiences.

### Respondents working in institutional care satisfied with information system support for CBKF

SWPs working in institutional care were the most satisfied with information system support for CBKF, whereas those working in the health care sector were the most dissatisfied. Professionals’ working environment impacts their information needs (Viitanen et al., 2022). Clients typically stay long term in social welfare institutional care and do not often change locations; these SWPs are likely to know these residents well and need less informational support from information systems. Moreover, residents’ needs are likely to remain relatively stable. In other services, SWPs need to rely more heavily on information systems for CBKF. For example, in healthcare, SWPs do not usually have access to the client’s documentation in the social welfare registry, whereas healthcare professionals’ documentation usually focuses on medical and nursing needs and thus does not support the needs and expectations of the professional role of SWPs. Moreover, SWPs are often not acknowledged as one of the end user groups of healthcare information systems, and their needs are not taken into account in system development (Salovaara et al., 2022c). Studies show that SWPs are willing to participate in the development of information systems, although participation methods need to be developed (Martikainen et al., 2022).

### Strengths and limitations

We were not able to determine the exact response rate; however, to the best of our knowledge, with 990 respondents, this survey was one of the largest on CIS usability in social welfare. Reliable statistical data on the number of highly educated SWPs from the data collection period are unavailable, which complicates the assessment of the representativeness of the respondent group. A strength of the survey is that it included several individual variables for each category: forming CBKF, usability, information quality and technical quality. The generalisability of the study results is further supported by the fact that respondents were distributed across employee sectors and service lines in a manner consistent with national reporting (Vuorijärvi et al., 2019). Results are consistent with the pilot study (Ylönen et al., 2020), which supports the reliability of the findings. One limitation of the study was the lack of information on which of the responding supervisors were engaged in client work and which were not. If a supervisor was not engaged in client work, they likely used the system mostly for information management purposes (Salovaara et al. 2023). The coefficient of determination for the multiple classification analysis model was 24.9%. As the objective was not to find all contributing factors, the result can be considered fairly good. In multiple classification analysis, it is recommended that the categories of independent variables be as equally sized as possible to enhance the reliability of the research findings. In this study, some categories of explanatory variables were of different sizes.

### Future research

In the future, field studies, including observations, are needed to investigate information systems support for CBKF and how user interfaces are designed and implemented. For example, transitioning from free-form documentation to structured frameworks represents a change for SWPs and needs to be investigated. Despite previous research, challenges remain in CISs, and it is essential to determine the reasons for this. Our results confirm that research on the usability of CISs remains important to support CIS development and improve the SWPs’ user experiences. In general, it is important to continue following the user experiences of CISs to monitor how different requirements guiding their development affect the usability, user experience, utility and perceived benefits of CISs. Future research should study user experiences in situations where an entire organisation transitions to a new CIS. Finland, in particular, provides an interesting research arena for the unique context of the development of a national architecture and integration between health and social welfare services, including information systems and information exchange. It is also essential to investigate whether professionals’ experiences with information systems differ based on the nature of their tasks, such as working in client guidance versus service delivery, where information needs may vary.

## Conclusion

Our research emphasises that CISs do not adequately support professionals for CBKF. They perceived CISs’ information quality to be good, but there was a need for improvement in usability. Usability and information quality were the strongest factors explaining SWPs’ experiences of information systems support for CBKF.

## Supplemental Material

sj-docx-1-him-10.1177_18333583251343681 – Supplemental material for Information system support for case-based knowledge formation in social welfare: a cross-sectional study Supplemental material, sj-docx-1-him-10.1177_18333583251343681 for Information system support for case-based knowledge formation in social welfare: a cross-sectional study by Elina Tynkkynen, Samuel Salovaara, Johanna Viitanen and Tinja Lääveri in Health Information Management Journal
