# Supplementary material for: Information system support for case-based knowledge formation in social welfare: a cross-sectional study
Source: Health Inf Manag. 2025 Jun 17;55(2):295–306. doi: 10.1177/18333583251343681 (PMC13187233; doi:10.1177/18333583251343681)
Supplement: sj-docx-1-him-10.1177_18333583251343681 – Supplemental material for Information system support for case-based knowledge formation in social welfare: a cross-sectional study [file sj-docx-1-him-10.1177_18333583251343681.docx]

# Appendix

*Table S1. Reclassified demographic data*

| Question | Original classification | Reclassification (combined categories) |
| --- | --- | --- |
| Age | 1 = Under 25, 2 = 25-34, 3 = 35-44, 4 = 45-54, 5 = 55-64, 6 = Over 64 | 1 = Under 35 (1,2), 2 = 35-44 (3), 3 = 45-54 (4), 4 = Over 54 (5,6) |
| What is your highest educational degree? | 1 = Licentiate or doctoral degree, 2 = Master degree 3 = Candidate degree, 4 = Master's degree from a university of applied sciences, 5 = Bachelor's degree, 6 = Lower Bachelor's degree, 7 = Other | 1 = Master's degree or higher (1,2,4), 2 = Bachelor's degree or equivalent (3,5,6,7) |
| Primary employment sector | 1 = Public, 2 = Private, 3 = Other (f.e. association) 4 = I dont know | 1 = Public sector (1), 2 = Private, association, other (2,3,4) |
| In which service line do you primarily work? | 1 = Services for families with children, 2 = Services for working age people, 3 = Services for elderly people, 4 = Family law services, 5 = Child welfare, 6 = Services for disabled people, 7 = Substance abuse services, 8 = Multiple service lines, 9 = Other work in social welfare 10 = School or corresponding | 1 = One service line (1,2,3,4,5,6,7), 2 = Multiple services lines (8), 3 = Other work in social welfare (9,10) |
| Job title in primary role | 1 = Manager or supervisor, 2 = Leading social therapist, 3 = Leading social worker, 4 = Trainer, teacher 5 = School social worker/responsible school social worker, 6 = Counsellor/responsible counsellor/coach, 7= Family counsellor, 8 = Project worker, 9 = Social counsellor, 10 = Social therapist, 11 = Social worker, 12 = Planner/coordinator, 13 = Researcher, 14 = Expert, 15 = Child supervisor, 16 = Family counsellor, 17 = Crisis worker, 18 = Criminal justice worker, 19 = Healthcare professional, 20 = Other | 1 = Supervisor (1,2,3) 2 = Social worker (11), 3 = Social counsellor (6,7,9), 4 = Other (4,5,8,10,12,13,14,15,16,17,18,19,20) |
| How long have you been using client information system? | 1 = Under ½ year, 2 = ½ year-under 1 year, 3 = 1 year-3 years, 4 = 3-6 years, 5 = over 6 years | 1 = Under 1y (1,2) 2 = 1y or more (3,4,5) |

*Table S2. Reclassified dependent variables*

| Question | Original classification | Reclassification (combined categories) |
| --- | --- | --- |
| When using client information system, do you primarily work in… | 1 = Open care services, 2 = Emergency work, 3 = Institutional care, 4 = Health care, 5 = Other | 1 = Open care services (1,2), 2 = Institutional care (3), 3 = Health care (4), 4 = Other (5) |
| What electronic client information system do you primarily use in your work? | 1 = Abilita, 2 = Apotti, 3 = ATJ/VATJ, 4 = Aura or AmmAura, 5 = DomaCare, 6 = Effica social care, 7 = Effica healthcare, 8 = Hilkka, 9 = Lifecare client and healthcare system, 10 = Mediatri, 11 = Nappula, 12 = Pegasos, 13 = Pro Consona, 14 = Sofia CRM, 15 = Uranus, 16 = ESKO-Oberon, 17 = Other, 18 = I don't use electronic client information system  (*9=Lifecare system refers only health care system)^16^ | 1 = Large CIS (1, 2, 3, 6, 10, 13), 2 = Focused CIS ((4, 5, 8, 11, 14) 3 = Patient information system (7, 9, 12, 15, 16) 4 = Other (17) |
